# Association of risk factors for high blood pressure across 46 low- and middle-income countries: A multi-country cross-sectional analysis

**DOI:** 10.7189/jogh.14.04087

**Published:** 2024-05-24

**Authors:** Yaping Hou, Shengan Yang

**Affiliations:** 1 Department of Emergency Medicine, Qilu Hospital of Shandong University, Jinan, Shandong, China; 2 Department of Logistics and Support Services, Qilu Hospital of Shandong University, Jinan, Shandong, China

## Abstract

**Background:**

Despite acknowledging the influence of various lifestyle and metabolic risk factors on hypertension, it remains uncertain to identify the primary contributors and differentiate which modifiable risk factors mediate the causal effects of hypertension. We aimed to examine the hierarchical association of eight prominent lifestyle and metabolic risk factors, along with demographic variables, with hypertension in adults and to explore the mediating effects of modifiable metabolic risk factors on hypertension.

**Methods:**

A cross-sectional study was conducted in 46 low- and middle-income countries using the World Health Organization (WHO) STEPwise approach to noncommunicable disease risk factor surveillance from 2002 to 2020. In a sample of 179 535 non-pregnant adults, we assessed the weighted population-attributable risk percentages (PAR%) for hypertension associated with eight risk factors. Additionally, we investigated the mediating role of metabolic risk factors on the effects of lifestyle risk factors on hypertension.

**Results:**

After adjusting for the sample weight in each country, 26.7% of participants had hypertension. The prevalence of hypertension was highest in those aged ≥65 years, with obesity-associated hypertension (45.7%) exceeding the rates for overweight (32.2%) and non-overweight individuals (18.2%). These eight risk factors collectively explain 83.7% of the PAR% associated with hypertension adjusted for the communal variance. Among the modifiable factors, obesity contributed to a weighted PAR% of 38.2%, while sedentary behaviour and low physical activity combined accounted for a weighted PAR% of 3.1%. Overweight/obesity played a predominant mediating role in the correlation between lifestyle risk factors and systolic and diastolic blood pressure, with the indirect effect accounting for approximately 25–64% and 13–80% of the total effect, respectively.

**Conclusions:**

These findings offer new insights into the modified risk factors associated with hypertension in adults in low- and middle-income countries, highlighting the crucial role of maintaining a normal body weight for the effective prevention and management of hypertension.

Hypertension is a primary global risk factor for mortality due to cardiovascular diseases, chronic kidney disease, and diabetes, contributing to more than 40% of deaths worldwide [1]. The burden of hypertension is notably pronounced in low- and middle-income regions, affecting more than one billion individuals, accounting for 82% of the global hypertensive population [2]. Importantly, hypertension tends to exhibit a faster rate of increase in low- and middle-income regions compared to high-income regions [2–4]. Additionally, awareness, treatment, and control rates for hypertension are significantly lower in low- and middle-income regions than in high-income regions [3].

The high prevalence of hypertension has led to a focus on addressing modifiable risk factors [5]. Recent research has identified several modifiable risk factors for hypertension, including socioeconomic determinants, overweight/obesity, physical inactivity/low fitness, unhealthy diet, smoking, alcohol consumption, and more [5,6]. However, there is still no clear consensus on the ranking of the effects of metabolic and lifestyle factors on hypertension. Establishing a definitive ranking of the contribution levels of hypertension risk factors could pave the way for a more precise and effective approach to hypertension management. Additionally, while substantial evidence suggests that hypertensive and obese individuals primarily need to maintain a normal body weight [7], research on the mediating effects of metabolic factors is limited.

To address this research gap, we estimated the population-attributable risk percentages (PAR%) of these identified risk factors with hypertension within 46 low- and middle-income regions, considering socioeconomic, lifestyle, and metabolic factors. Additionally, we investigated the mediating effect of metabolic risk factors between lifestyle risk factors and hypertension, offering new insights for preventing high blood pressure.

## METHODS

### Study design and participants

We obtained the data from a cross-sectional population-based study using the World Health Organization (WHO) STEPwise approach to noncommunicable disease risk factor surveillance (STEPS) from 2002 to 2020. We employed a direct and standardised approach to collect, analyse, and disseminate data on crucial noncommunicable disease risk factors in various countries [8]. We encompassed key behavioural risk factors, including tobacco use, alcohol consumption, physical inactivity, and unhealthy diet, as well as critical biological risk factors, such as overweight/obesity and raised blood pressure. All participating countries utilised the same standardised questions and protocols.

We conducted a secondary analysis of the primary data, including countries with relatively complete demographic information, tobacco use, alcohol consumption, diet, physical activity, body mass index (BMI), and blood pressure. We investigated data from 46 low- and middle-income countries, involving 179 535 adults with a mean age of 37 (range 18–108). During the STEPwise study, all procedures were conducted following predetermined instructions, and written informed consent was obtained from all participants.

### Data collection

#### Outcome

Blood pressure (BP) was measured three times after 15 minutes of rest, and the average reading of the last two BP readings was used to define hypertension. We determined the hypertensive status of the subjects based on the 2018 hypertension guidelines for managing arterial hypertension in adults, published jointly by the European Society of Cardiology and the European Society of Hypertension. The criteria were systolic blood pressure (SBP)≥140mmHg and/or diastolic blood pressure (DBP)≥90mmHg or taking antihypertensive medication [9]. In 2017, the American College of Cardiology/American Heart Association task force on clinical practice guidelines redefined hypertension in adults as SBP≥130 mm Hg and DBP≥80 mm Hg, and using antihypertensive drugs [7]. To avoid confusion caused by different diagnostic criteria for hypertension, we have included the analysis of the 2017 American College of Cardiology/American Heart Association diagnosis of hypertension as well (Table S1 in the **Online Supplementary Document**).

#### Metabolic and lifestyle factors

Participants’ weight and height were measured using standardised techniques to estimate BMI (kg/m^2^) [10]. The BMI was classified as non-overweight (<25 kg/m^2^), overweight (25–30 kg/m^2^), and obese (≥30 kg/m^2^) [10]. We characterised low fruit and vegetable intake as consumption of less than five servings of fruit and vegetables per day, as recommended by the WHO [11]. Cigarette smoking status was classified into two categories – current or former smokers and never smokers. Alcohol consumption was categorised into current or former drinkers and non-drinkers. Sedentary time was defined as the duration of sitting for over 240 minutes [12]. Physical activity behaviour was evaluated across three domains – work, transportation, and leisure. We classified activity into inactive, moderate, and vigorous. The metabolic equivalent of task (MET) represents the ratio of metabolic rate during activity to their resting metabolic rate. The assessment of physical activity level involved determining the frequency and intensity of physical activity per week to calculate MET minutes per week. Insufficient physical activity was defined as less than 600 MET minutes per week. Moderate physical activity ranged from 600–3000 MET minutes per week, while vigorous physical activity exceeded 3000 MET minutes per week [11,13,14]. We obtained these data through self-reporting.

#### Statistical analysis

We used the multiple imputation by chained equations method to mitigate potential bias due to missing data [15,16]. This technique handles missing data by iteratively imputing values for each missing variable based on the observed values of other variables in the data set. Data imputation was performed for all variables with missing information, including BMI (0.7% missing), low fruit and vegetable intake (2.9% missing), smoking (0.04% missing), alcohol use (0.2% missing), physical activity (11.8% missing), and sedentary behaviour (3.6% missing). Additionally, we included complete variables such as age, sex, and hypertension in the imputation process, resulting in the generation of five imputed data sets for subsequent analysis.

To assess the normality of the continuous variables, we performed a normality test using the Kolmogorov-Smirnov test and Quantile-Quantile plot. We summarised baseline participants’ characteristics as weight-adjusted means with standard errors for continuous variables and unadjusted original counts and weight-adjusted percentages for categorical variables. Further, we assessed differences in baseline characteristics using weighted linear regression for continuous variables and the Rao-Scott χ^2^ test for categorical variables. We employed a bar graph to represent the weight-adjusted prevalence of hypertension among different participants visually. We used the weighting to enhance the representativeness of the sample and the ability to generalise to the whole population.

Further, we employed multivariate logistic regression models to calculate odds ratios (ORs) and confidence intervals (CIs) for the risk of hypertension associated with risk factors while adjusting for sample weights. Multicollinearity diagnostics indicated an absence of collinearity between individual risk factors (Methods S1 in the **Online Supplementary Document**).

Using a well-established partial PAR method, we calculated PAR%, which represents the proportion of the cases in our study population that could potentially be prevented if no one had been exposed to specific risk factors, individually or in combination [17]. PAR% were calculated using the results generated by the PAR macro developed by Ellen Hertzmark, Handan Wand, Donna Spiegelman, and their collaborators [18,19] (Method S2 in the **Online Supplementary Document**).

We analysed the mediating effects of overweight/obesity using regression-based methods. This involved breaking down the overall effects of lifestyle risk factors on SBP and DBP into total, direct, and indirect effects. The proportion mediated by overweight/obesity was calculated accordingly [18–20]. All statistical analyses were performed with SAS, version 9.4 (SAS Institute, Cary, North Carolina, USA). A two-tailed *P*-value <0.05 was established as statistical significance.

## RESULTS

### Participants’ characteristics

**Figure 1:**
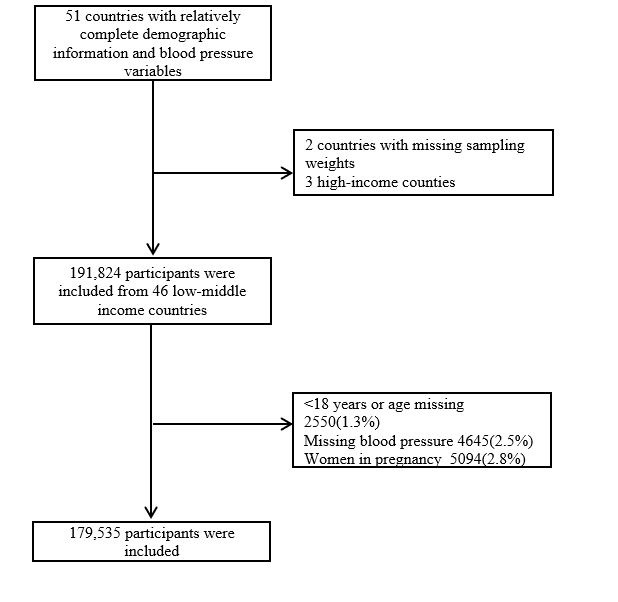
Flowchart for study inclusion.

After a thorough screening process to ensure completeness of variables, excluding records from children, adolescents, pregnant women, and those with missing blood pressure measurements, a total of 179 535 adults actively participated in the study (**Figure 1**). Among them, 105 935 were females with a mean age of 37.4 years, while 73 600 were males with a mean age of 36.6 years. Statistically significant differences were found in age, BMI, SBP, DBP, overweight, obesity, smoking, alcohol consumption, and physical activity between males and females. However, there was no gender difference in the prevalence of hypertension, which was 27.0% in males and 26.4% in females (**Table 1**).

**Table 1:** Baseline characteristics of participants after adjusting sampling weights*

Characteristics	Female (n = 105 935)	Male (n = 73 600)	Total (n = 179 535)	*P*-value
Age in years, x̄ (SE)	37.4 (0.09)	36.6 (0.11)	37.0 (0.07)	<0.0001†
Age group				<0.0001
*18–24*	11 107 (19.8)	8278 (22.3)	19 385 (21.1)	
*25–34*	27 577 (28.1)	18 764 (28.7)	46 341 (28.4)	
*35–44*	25 199 (21.6)	17 279 (21.1)	42 478 (21.3)	
*45–54*	21 404 (16.9)	14 990 (14.5)	36 394 (15.7)	
*55–64*	16 981 (10.6)	11 777 (10.1)	28 758 (10.3)	
*≥65*	3667 (3.1)	2512 (3.3)	6179 (3.2)	
Height in cm**)**, x̄ (SE)	158.2 (0.05)	169.8 (0.06)	164.2 (0.05)	<0.0001†
Weight in kg**)**, x̄ (SE)	65.2 (0.11)	70.5 (0.13)	67.9 (0.08)	<0.0001†
BMI in kg/m2), x̄ (SE)	26.1 (0.04)	24.4 (0.04)	25.2 (0.03)	<0.0001†
SBP in mmHg), x̄ (SE)	124.5 (0.13)	128.3 (0.14)	126.5 (0.09)	<0.0001†
DBP in mmHg), x̄ (SE)	79.9 (0.08)	80.2 (0.09)	80.1 (0.06)	0.0889†
Weight				<0.0001
*Non-overweight*	47 963 (50.1)	42 138 (61.3)	90 101 (55.9)	
*Overweight*	28 719 (26.8)	19 928 (26.3)	48 647 (26.5)	
*Obesity*	29 253 (23.2)	11 534 (12.4)	40 787 (17.6)	
Low fruit and vegetable intake				0.7327
*No*	10 412 (7.6)	7360 (7.6)	17 772 (7.6)	
*Yes*	95 523 (92.4)	66 240 (92.4)	161 763 (92.4)	
Smoke				<0.0001
*No*	95 910 (86.0)	38 814 (47.9)	134 724 (66.3)	
*Yes*	10 025 (14.0)	34 786 (52.1)	44 811 (33.7)	
Alcohol use				<0.0001
*No*	67 525 (74.3)	33 633 (61.8)	101 158 (67.8)	
*Yes*	38 410 (25.7)	39 967 (38.2)	78 377 (32.2)	
Physical activity				<0.0001
*Inactive*	24 455 (24.9)	11 974 (14.7)	36 429 (19.6)	
*Moderate*	27 108 (27.2)	14 470 (20.8)	41 578 (23.9)	
*Vigorous*	54 372 (47.9)	47 156 (64.5)	101 528 (56.5)	
Sedentary				0.6429
*No*	64 866 (59.6)	45 020 (59.9)	109 886 (59.7)	
*Yes*	41 069 (40.4)	28 580 (40.1)	69 649 (40.3)	
Hypertension				0.1895
*No*	73 907 (73.6)	50 398 (73.0)	124 305 (73.3)	
*Yes*	32 028 (26.4)	23 202 (27.0)	55 230 (26.7)	

BMI – body mass index, DBP – diastolic blood pressure, SE – standard error, SBP – systolic blood pressure, x̄ – mean

*Presented as n (%) unless specified otherwise. N are unadjusted original counts, and the percentages and means (standard errors) are sample-adjusted values.

†*P*-values from weighted linear regression. Other *P*-values are from the Rao-Scott χ^2^ test, with adjustment for sample weights.

### Adjusted prevalence of hypertension

**Figure 2:**
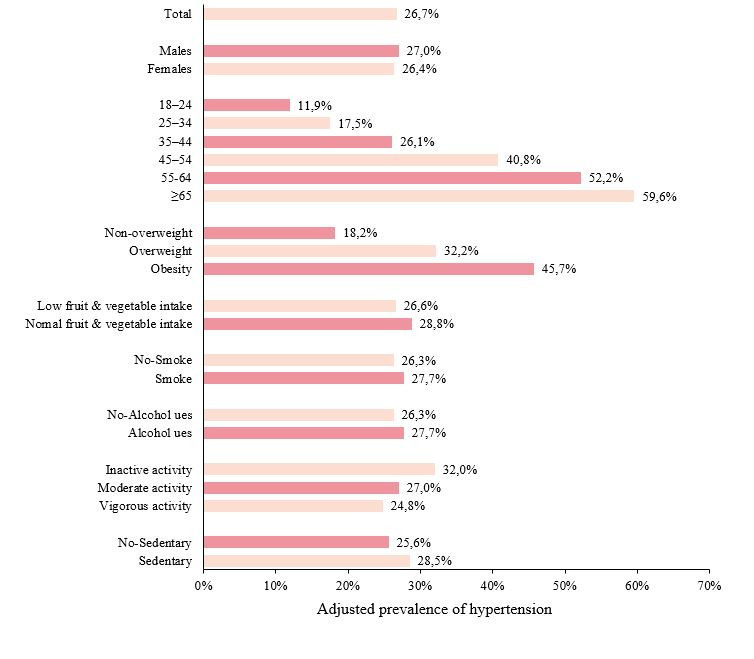
Weighted prevalence of hypertension in different subgroups.

After adjusting for sampling weights, the overall prevalence of hypertension among adults was determined to be 26.7%. Notably, the prevalence of hypertension within each subgroup exhibited significant statistical variation (*P* < 0.0001). There was a trend for the prevalence of hypertension to increase with age. Specifically, the prevalence of hypertension was 32.2% in overweight adults and 45.7% in those classified as obese. Additionally, suboptimal fruit and vegetable consumption, tobacco use, alcohol consumption, insufficient physical activity, and sedentary behaviour were all associated with higher prevalence rates of hypertension (**Figure 2**).

### Multivariate analysis of hypertension risk factors

In a multivariate analysis, accounting for adjusted sampling weights, males had a higher risk of hypertension than females (OR = 1.30; 95% CI = 1.22, 1.38). Furthermore, the risk of hypertension demonstrated an upward trend with advancing age, with significantly increased risk observed among older age groups. Specifically, adults classified as obese (OR = 2.89; 95% CI = 2.71, 3.08) and those categorised as overweight (OR = 1.73; 95% CI = 1.63, 1.84) had a significantly increased risk of hypertension (**Table 2**). Notably, to investigate the impact of various hypertension diagnostic criteria on our findings, we incorporated the 2017 American College of Cardiology/American Heart Association hypertension diagnosis and the results were consistent (Table S1 in the **Online Supplementary Document**).

**Table 2:** Weight-adjusted multivariable OR (95% CI) for hypertension (SBP/DBP≥140/90) associated with individual risk factors among overall participants

Characteristics	Univariate OR (95% CI)	*P*-value*	Multivariate OR (95% CI)	*P*-value†
Sex				
*Female*	ref.		ref.	
*Male*	1.03 (0.99, 1.08)	0.1893	1.30 (1.22, 1.38)	<0.0001
Age group				
*18–24*	ref.		ref.	
*25–34*	1.57 (1.41, 1.74)	<0.0001	1.41 (1.27, 1.57)	<0.0001
*35–44*	2.62 (2.36, 2.90)	<0.0001	2.16 (1.95, 2.40)	<0.0001
*45–54*	5.10 (4.62, 5.62)	<0.0001	4.07 (3.67, 4.50)	<0.0001
*55–64*	8.08 (7.28, 8.97)	<0.0001	6.35 (5.70, 7.07)	<0.0001
*≥65*	10.91 (9.44, 12.61)	<0.0001	8.76 (7.54, 10.17)	<0.0001
Weight				
*Non-overweight*	ref.		ref	
*Overweight*	2.13 (2.01, 2.25)	0.0005	1.73 (1.63, 1.84)	0.4756
*Obese*	3.77 (3.55, 4.01)	<0.0001	2.89 (2.71, 3.08)	<0.0001
Low fruit and vegetable intake				
*No*	ref.		ref.	
*Yes*	0.90 (0.83, 0.97)	0.0037	0.95 (0.87, 1.03)	0.1907
Smoke				
*No*	ref.		ref.	
*Yes*	1.08 (1.02, 1.14)	0.0087	0.91 (0.85, 0.97)	0.0071
Alcohol use				
*No*	ref.		ref.	
*Yes*	1.07 (1.03, 1.12)	0.0024	0.95 (0.91, 1.00)	0.0653
Physical activity				
*Inactive*	ref.		ref.	
*Moderate*	0.79 (0.73, 0.84)	0.0269	0.92 (0.86, 1.00)	0.0570
*Vigorous*	0.70 (0.66, 0.75)	<0.0001	0.96 (0.90, 1.03)	0.9295
Sedentary				
*No*	ref.		ref.	
*Yes*	1.16 (1.10, 1.22)	<0.0001	1.05 (0.99, 1.10)	0.0902

CI – confidence interval, OR – odds ratio, ref – reference

*P-values from univariate logistic regression models, adjusted for sample weights.

†*P*-values from multivariate logistic regression models, adjusted for sample weights.

### Weighted PAR% of hypertension risk factors

**Figure 3:**
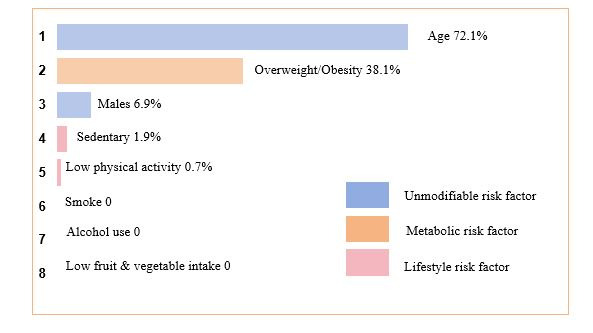
Weighted population-attributable risk percentages for hypertension associated with risk factors. PAR% illustrates the portion of hypertension that could have been avoided if individuals had not been exposed to specific risk factors in this study after adjustment for the communal variance. Individual risk factors with negative PAR% were not included in these analyses. The negative PAR% was truncated at the lower limit of zero, as this was the lowest threshold to detect an association with increased risk. PAR% – population-attributable risk percentages.

In terms of the PAR%, age emerged as the primary risk factor for hypertension in adults, followed by overweight/obesity, gender, sedentary lifestyle, and low physical activity. The overall PAR% for hypertension was 83.7%, adjusted for the shared variance among each included risk factor. In addition to unmodifiable risk factors such as gender and age, metabolic risk factors-specifically, overweight and obesity stood out as the most influential contributors to hypertension, accounting for a substantial weighted PAR% = 38.1% (**Figure 3**). The cumulative weighted PAR% for lifestyle-related risk factors associated with hypertension was 2.6%, markedly lower than that for metabolic risk factors.

### Mediation analysis of lifestyle factors on blood pressure through BMI

**Figure 4:**
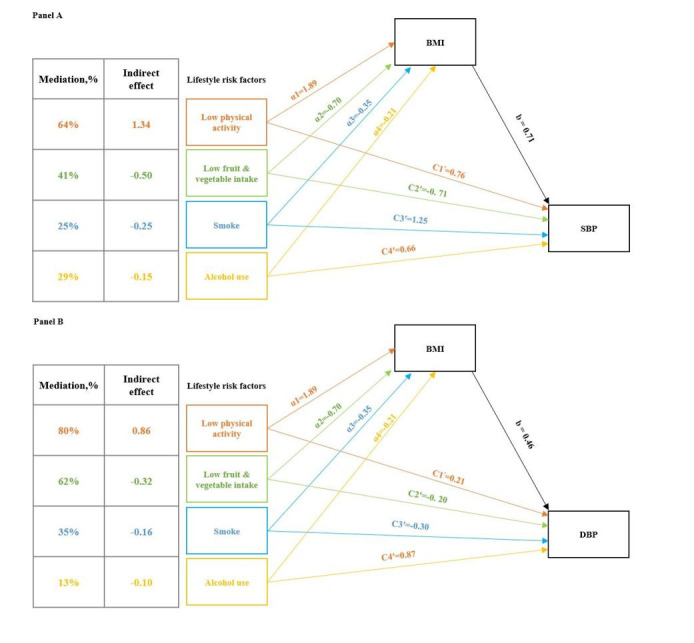
Mediation analysis to determine the relationship between lifestyle risk factors and SBP/DBP through BMI. **Panel A**. The outcome of the mediation analysis is SBP. **Panel B**. The outcome of the mediation analysis is DBP. α1, α2, α3, and α4 represent the effects of low physical activity, low fruit and vegetable intake, smoking, and alcohol use on BMI, respectively. The parameter ‘b’ represents the effect of BMI on SBP/DBP. C1^′^, C2^′^, C3^′^, and C4^′^represent the direct effects of low physical activity, low fruit and vegetable intake, smoking, and alcohol use on SBP/DBP, respectively. Total effects consist of the sum of indirect and direct effects. The mediation percentage is calculated by dividing the indirect and total effects. All effects have *P* < 0.05. Sedentary was not included in the analysis as its total effects on SBP and DBP were non-significant (*P* > 0.05). BMI – body mass index, DBP – diastolic blood pressure, SBP – systolic blood pressure.

In the mediation analysis, lifestyle risk factors exhibited direct effects on blood pressure and exerted indirect effects through BMI on blood pressure. The mediation indirect effects of low physical activity, low fruit and vegetable intake, smoking, and alcohol use via BMI on SBP were 64, 41, 25, and 29%, respectively. For DBP, the mediation indirect effects of low physical activity, low fruit and vegetable intake, smoking, and alcohol use via BMI were 80, 62, 35, and 13%, respectively (**Figure 4**).

## DISCUSSION

This cross-sectional study, conducted across 46 low- and middle-income countries, provided valuable insights into the relationship between modifiable risk factors and adult hypertension. Our study highlights that overweight/obesity is the foremost modifiable risk factor for hypertension in adults, followed by factors such as a sedentary lifestyle and low physical activity. These factors collectively account for a significant portion of the overall risk of hypertension. Additionally, lifestyle-related risk factors associated with hypertension collectively account for a smaller proportion of the risk, emphasising the complexity of the aetiology of hypertension. In the mediation analysis, high BMI partially mediates the relationship between lifestyle risk factors and blood pressure, further highlighting the interconnected nature of these factors in the development of hypertension. These findings emphasise the importance of addressing metabolic risk factors, particularly overweight and obesity, in hypertension prevention and management strategies while recognising the role of lifestyle factors in influencing blood pressure levels.

The role of overweight and obesity as risk factors for hypertension was more significant than several lifestyle risk factors. Obesity and hypertension are closely linked because belly fat interferes with the endocrine and immune systems, increasing the risk of insulin resistance, diabetes, hypertension, and cardiovascular disease [21–23]. For instance, findings from the Nurse’s Health Study showed that women who gained 5–10 kg and >25 kg experienced a relative risk of hypertension of 1.7 and 5.2, respectively. This study further revealed that 40% of new-onset hypertension cases were attributed to overweight and obesity [24]. It is a key recommendation from the 2018 European Society of Cardiology/European Society of Hypertension and 2017 American College of Cardiology/American Heart Association guidelines on non-pharmacological intervention for hypertension that overweight or obese adults with elevated blood pressure or hypertension should aim for weight loss to reduce their blood pressure effectively [7,9]. Corroborating this, a systematic review and meta-analysis encompassing 25 randomised controlled trials with 4874 participants found that each one kg reduction in body weight was associated with an average decrease in SBP of 1.05 mm Hg (95% CI = –1.43, –0.66) [25].

Our research indicates that sedentary behaviour and low physical activity, which are integral parts of an unhealthy lifestyle, independently elevate the risk of hypertension. This finding is consistent with similar results from other studies. A meta-analysis of 27 randomised controlled trials has shown that medium-to-high-intensity aerobic exercise significantly lowers blood pressure in hypertensives by an average of 11/5 mm Hg, with isometric and dynamic resistance exercises showing comparable effects [26]. In a controlled study, the highest sedentary time category correlated with a 34% increased hazard ratio for heart failure compared to the lowest, independent of other risk factors [27]. Additionally, a cohort study demonstrated linear dose-response relationships between sedentary time and bout duration and cardiovascular disease risk [28]. A multifaceted approach was often most effective regarding non-pharmacological management of hypertension, combining strategies like maintaining a healthy weight, nutritious diet, and regular exercise. Remarkably, individuals with a healthy lifestyle, as determined by a composite score that includes factors such as diet, smoking status, drinking status, physical activity, and BMI, exhibited significantly lower blood pressure levels and a reduced risk of hypertension compared to those with an unhealthy or intermediate lifestyle [29].

Our study demonstrates that lifestyle risk factors affect hypertension through the mediating effect of obesity. This finding is consistent with the results of other studies. Min Yuan’s study showed that BMI and waist-to-height ratio are entirely mediated between the dietary approaches to stop hypertension diet score and hypertension levels. Together, these factors accounted for over 45% of the variation in hypertension [30]. However, there is limited research exploring the mediating role of metabolic influencing factors between lifestyle risk factors and hypertension. A recent study using summary statistics from genome-wide association studies of predominantly European ancestry found that education, intelligence, and cognition affect hypertension and blood pressure through the mediating effects of modifiable cardiometabolic factors [31]. Metabolic risk factors seem to play a significant role among the various risk factors for hypertension.

This is one of the few large studies involving 46 low- and middle-income countries to quantify common modifiable risk factors for hypertension in adults. Key strengths of our study were the large sample size and reliable risk factor measurement of risk factors on a global scale. It is also essential to be aware of potential limitations. First, this study is cross-sectional and cannot assess the risk of incident hypertension, which limits causal inference. Second, although we carefully controlled for confounders in our analysis, there may be bias from unmeasured confounders and reverse causality. For example, salt intake is an important risk factor for hypertension and interacts with other lifestyle and metabolic risk factors.

## CONCLUSIONS

This study clarified the independent effects of metabolic and lifestyle factors on hypertension. It ranked the modifiable risk factors for hypertension according to weighted PAR%, with overweight/obesity, sedentary behaviour and low physical activity ranked in order. The study also showed that metabolic factors play a significant mediating role in the influence of lifestyle factors on blood pressure. These findings provide causal evidence for the aetiology of hypertension and support the development of tailored approaches to primary prevention and management strategies for hypertension.

## Additional material


Online Supplementary Document
