# Supplementary material for: Association of risk factors for high blood pressure across 46 low- and middle-income countries: A multi-country cross-sectional analysis
Source: J Glob Health. 2024 May 24;14:04087. doi: 10.7189/jogh.14.04087 (PMC11117091; doi:10.7189/jogh.14.04087)
Supplement: Online Supplementary Document [file jogh-14-04087-s001.pdf]

Supplementary Document

Table S1. Weight-adjusted multivariable odds ratios (95% CIs) for hypertension (SBP/DBP≥ 130/80) associated with individual risk factors among overall participants

	N	%	Univariate OR (95%CI)	*P*-value‡	Multivariate OR (95%CI)	*P*-value§
**Sex**						
Females	58770	53.86	Reference		Reference	
Males	44613	58.58	1.21(1.16-1.27)	<0.0001	1.59(1.51-1.68)	<0.0001
**Age group**						
18–24	6787	40.95	Reference		Reference	
25–34	20540	48.96	1.38(1.29-1.49)	<0.0001	1.29(1.2-1.38)	<0.0001
35–44	23922	58.41	2.03(1.88-2.18)	<0.0001	1.76(1.63-1.91)	<0.0001
45–54	25002	69.40	3.27(3.03-3.53)	<0.0001	2.77(2.56-3.00)	<0.0001
55-64	22179	76.69	4.75(4.35-5.17)	<0.0001	3.89(3.55-4.25)	<0.0001
≥65	4953	78.61	5.30(4.51-6.23)	<0.0001	4.27(3.62-5.04)	<0.0001
**Weight**						
Non-overweight	42906	46.57	Reference		Reference	
Overweight	30630	64.54	2.09(1.98-2.20)	<0.0001	1.81(1.72-1.91)	0.0079
Obese	29847	74.87	3.42(3.21-3.64)	<0.0001	2.84(2.65-3.04)	<0.0001
**Low fruit & vegetable intake**						
No	10618	58.15	Reference		Reference	
Yes	92765	56.16	0.92(0.86-0.99)	0.0268	0.96(0.89-1.04)	0.3489
**Smoke**			Reference			
No	77411	56.00	Reference		Reference	
Yes	25972	56.92	1.04(0.98-1.09)	0.1682	0.83(0.78-0.88)	<0.0001
**Alcohol use**						
No	58040	56.88	Reference		Reference	
Yes	45343	55.11	0.93(0.89-0.97)	0.0007	0.84(0.80-0.88)	<0.0001
**Physical activity**						
Inactive activity	22173	62.92	Reference		Reference	
Moderate activity	24026	56.20	0.76(0.71-0.81)	0.0004	0.85(0.79-0.91)	0.0045
Vigorous activity	57184	54.06	0.69(0.66-0.73)	<0.0001	0.85(0.80-0.91)	0.0024
**Sedentary**						
No	62932	54.97	Reference		Reference	
Yes	40451	58.29	1.14(1.09-1.20)	<0.0001	1.07(1.02-1.12)	0.0074

OR – odds ratio, 95%CI – 95% confidence interval.

Ns represents the unadjusted raw count of hypertension cases, whereas %(percentage) represents the adjusted prevalence of hypertension within each group after sample adjustments.

‡*P*-values from univariate logistic regression models, adjusted for sample weights.

§*P*-values from multivariate logistic regression models, adjusted for sample weights.

Method S1. Diagnosis of multicollinearity

Step 1: The correlation matrix between each risk factor was examined using the SAS PROC CORR procedure (Kendall's tau-b correlation coefficients). The correlation coefficients ranged from -0.15 to 0.43. After reviewing the correlation matrix, there appear to be no variables with a particularly high correlation (coefficient 0.8 or higher)[1].

**Correlation coefficients**								
Variables	gender	age group	Ov/Ob	diet	smoke	alcohol	activity	sedentary
gender	1	-0.00	**-0.15**	-0.00	**0.43**	0.18	0.12	-0.00
age group	-0.00	1	0.23	-0.02	0.05	0.03	-0.08	0.02
Ov/Ob	**-0.15**	0.23	1	-0.03	-0.03	-0.03	-0.14	0.06
diet	-0.00	-0.02	-0.03	1	0.02	-0.01	-0.03	-0.02
smoke	**0.43**	0.05	-0.03	0.02	1	0.21	0.03	0.06
alcohol	0.18	0.03	-0.03	-0.01	0.21	1	0.11	-0.01
activity	0.12	-0.08	-0.14	-0.03	0.03	0.11	1	-0.18
sedentary	-0.00	0.02	0.06	-0.02	0.06	-0.01	-0.18	1

Ov/Ob=overweight/obesity, diet=low fruit & vegetable intake.

Step 2: To examine multicollinearity through the variance inflation factor, and for "variance inflation", no values above 10. Therefore, the data of multicollinearity diagnosis indicated a lack of multicollinearity of these variables[1].

**Variance Inflation**	
gender	1.28
age group	1.06
overweight/obesity	1.09
diet	1.00
smoke	1.27
alcohol	1.07
activity	1.08
sedentary	1.04

diet=low fruit & vegetable intake.

References:

1. Paul D. Allison. Logistic Regression Using SAS: Theory and Application, Second Edition. 2012. SAS Institute Inc. SAS Campus Dr. Cary, NC, United States.

Method S2 Calculation formula for the population-attributable risk percentage

In this study, we assessed the population-attributable risk percentages (PAR%) and their corresponding 95% confidence intervals (95% CIs) for hypertension, considering the influence of individual risk factors and their combinations. The PAR is calculated based on the relative risk(s) and the prevalence(s) of the risk factor(s), providing a measure of the proportion of cases of a certain outcome attributable to the presence of those factors in the population.

$PAR = \frac{p(RR-1)}{p(RR-1)+1}$, where RR is the relative risk and P is the prevalence of the exposure in the population.

In the study, we applied the partial PAR method, developed by Ellen Hertzmark, Handan Wand, and Donna Spiegelman[1,2], which accounts for the impact of risk factor(s) on an outcome while adjusting the influence of other variables. Notably, we excluded negative PAR% values from our models, and these were adjusted to a minimum threshold of 0. This threshold was chosen because it signifies the point at which an association with an increased risk begins to emerge.

References:

1. Spiegelman D, Hertzmark E, Wand HC. Point and interval estimates of partial population attributable risks in cohort studies: examples and software. Cancer Causes Control 2007; 18:571-9.
2. Hertzmark E, Wand H, Spiegelman D. The SAS PAR Macro. 2012. Available: https://www.hsph.harvard.edu/donna-spiegelman/software/par/. Accessed: 10 March 2024.
